# Abundance and diversity of cotton insect pests under repeatedly cultivated cotton fields of Tanzania

**DOI:** 10.3389/finsc.2024.1385653

**Published:** 2024-09-18

**Authors:** Madama Benjamin, Gration M. Rwegasira

**Affiliations:** Department of Crop Science and Horticulture, Sokoine University of Agriculture, Chuo Kikuu, Morogoro, Tanzania

**Keywords:** pests abundance, cotton, diversity, species richness, continuous repeated cotton farming

## Abstract

**Introduction:**

Cotton production in Tanzania is facing significant challenges due to insect pests that cause extensive damages to the crop. The most notable pests include the African bollworm (*Heliothis armigera* Hubner), Spiny bollworm (*Earias biplaga* Walker), Cotton stainers (*Dysdercus sidae* (Herrich-Schaeffer), Cotton Aphids (*Aphis gossypii* Glover), Thrips (*Thrips tabaci* Lindeman), Jassids (*Amrasca biguttula*, Bigutula), Leafhoppers (*Cicadellidae jassidae*), and Whiteflies (*Bemisia tabaci* Genn). If left uncontrolled, these pests can cause up to 60% damage to the crop. Despite the importance of cotton and the fact that most of these pests are endemic, there are scanty knowledge on the dynamics and distribution of cotton pests across the seasons of the year and crop’s phenological growth stages (germination, vegetative growth, flowering and boll formation) in areas under repeated cultivation of the crop in Tanzania. Here we report on the influence of seasons and cotton’s phenological stages on the abundance, diversity, distribution and richness of cotton insect pests.

**Methods:**

The study was conducted in the Misungwi district for two cotton-growing seasons, using the UKM08 cotton variety. Stick traps and handpicking methods were deployed in catching the cotton insect pests.

**Results:**

On average, a total of 8,500 insect specimen of diverse families and species were collected every season. The four dominant species among the collected were *Aphis gossypii* (17.37%), *Amrasca biguttula* (11.42%), *Nezara viridura* (10.7%), and *Bemisia tabacci* (10.68%). Both cotton phenological growth stages and seasons significantly (*p*<0.05) influenced the abundance, diversity, distribution and richness of cotton insect pests. In particular, the phenological growth stage 3 exhibited greater diversity of insect pests. The pests’ distribution patterns remained relatively uniform across the crop growth stages.

**Discussion:**

Findings from the present study could contribute to developing sustainable pest management strategies in areas under repeated cotton production in Tanzania and elsewhere.

## 1 Introduction

Cotton (*Gossypium hirsutum* L) production in Tanzania is severely constrained by insect pests that causes significant damage to the crop. Both Western and Eastern cotton-growing areas are equally affected (1). Mrosso et al. (2) indicated that the most harmful cotton insect pests in Tanzania includes, the African bollworm (*Heliothis armigera* Hubner), Spiny bollworm (*Earias biplaga*), Cotton stainers (*Dysdercus cingulatus*), Cotton Aphids (*Aphis gossypii*), Thrips (*Thrips tabaci*), Jassids (*Amrasca biguttula*), and Leafhoppers (*Cicadellidae jassidae*). If left unchecked, these pests can cause up to 60% damage to cotton crops (2, 3).

Cotton crops faces significant yield losses due to sucking and chewing insect pests, with record losses of up to 16.55 and 17.35 quintal ha^−1^, respectively (4). Of the total agricultural losses inflicted by insect pests, cotton alone accounts for 84%. For example, off all the insects, Jassid is known to cause hazardous effects up to 18.78% decline in cotton yield (5). Similarly, in Pakistan in 1993, the whitefly vector of CLCuV injured cotton by secreting honeydew and transmitting cotton leaf curl viral diseases causing up to 38.7% yield loss (6, 7).

Despite the critical importance of the cotton crop in Tanzania and the significant economic losses incurred due to insect pests (8) there is a lot of unknown regarding the pest dynamics. Studies exploring the influence of cotton phenological growth stages and seasons on insect pests infesting the crop are scarce particularly in areas under repeated cotton cultivation. Studies elsewhere have focused on the composition of insect pests in a cotton crop and their variation through the cotton phenological growth stages and cropping seasons. (3). Understanding the influence of these factors on the cotton pest dynamics is crucial for developing effective pest management strategies and safeguarding the productivity of the crop in Tanzania.

It is imperative to understand how insect pest composition varies across different growth stages (germination, vegetative growth, flowering and boll formation) and seasons in order to devise targeted and season-specific pest management strategies. The economic significance of the cotton crop, both nationally and globally, amplifies the importance of such research (9). Furthermore, the prevalence of factors such as high polyphagy, wide geographical range, mobility, migratory potential, facultative diapause, high fecundity, and resistance to insecticides that contribute to the pest status of these insects emphasizes on the need for nuanced and context-specific interventions.

Therefore, the objectives of this study were i) to determine the abundance and richness of cotton insect pests as influenced by the crop’s phenological developmental stages, ii) to establish the diversity and distribution of cotton pests as influenced by season dynamics. Here we report on the cotton pest dynamics in areas under repeated production of the crop in the Western Cotton Growing Areas of Tanzania. The study findings will contribute to development of sustainable and effective pest management strategies in the area for improved returns from investments in cotton.

## 2 Materials and methods

### 2.1 Study site

The study was conducted for two cotton-growing seasons, namely “Season I” (November 2021 to May 2022) and “Season II” (November 2022 to May 2023), at Misungwi district in Mwanza, Tanzania. The study site was located at S 02°43.1′ and E033°1.0′, approximately 30 km from Mwanza City at an altitude of 1198 m above sea level (10). The mean annual rainfall in the area was about 930 mm, and the average temperature was 28°C. The region experiences a bimodal rainfall pattern, with short rains falling from October to December and long rains from March to May. Following the rainy seasons, there is usually a dry spell from June to September each year (9). Misungwi district is located within the Western Cotton-Growing Areas in Tanzania.

### 2.2 Study materials

The materials used in the experiment were stick traps and cotton seeds. The cotton seeds variety UKM08 was obtained from the Tanzania Agricultural Research Institute (TARI-Ukiriguru). The UKM08 variety was chosen for its high yield and good cotton fibre characteristics. The variety is commercially cultivated throughout Tanzania in the Western and Eastern Cotton Growing Areas.

### 2.3 Experimental design

The experiment was set in a split plot in Randomized Complete Block Design (RCBD) with three replications. The main factors were seasons (Seasons 1 and 2), and the sub-factors were cotton phenological growth stages (germination, vegetative growth, and flowering and boll formation as stages 1, 2 and 3). The field experiments were carried out from November 2021 to May 2022 and then repeated from November 2022 to May 2023. The aim of the study was to determine the abundance, richness, distribution and diversity of insect pests in cotton fields under repeated cultivation of cotton. The experiments were conducted in three fields to make up three replications, each measuring 6,574m² (173m by 38m) established at least 50m apart from each other. Each field (replication) was divided into three subplots measuring 2,052m² (54m by 38m) each, separated by a 4m wide gap left between consecutive plots with a guard row measuring 1.5 wide on each field margin. Cotton seeds of the UKM08 variety were sown at a spacing of 0.6m by 0.3m then thinned to allow one plant per stand. The crops were maintained under rain-fed conditions and agronomic practices were implemented as per standard recommendations, including weed management and fertilizer application (manure) but no pesticide was applied.

### 2.4 Data collection of insect pests

Sampling of insect pests began 26 days after germination. For each subplot, twenty plants were randomly selected and marked with plastic tags. The sampling of insect pests was carried out on each tagged plant on a weekly basis for three consecutive weeks in each of the cotton growth stages: first square (between 27 and 56 days from germination), second square (between 57 and 82 days) and third square (between 83 and 145 days). This was done over two cotton growing seasons, from November 2021 to May 2022 as season one and from November 2022 to May 2023 as season two.

Two sampling methods were considered depending on the nature of feeding of respective pest groups that is, insects which suck and chew on the leaves and bolls of cotton whereby sticky traps (red, green and yellow) and hand-picking on three units measuring 9m × 54 m = 486m^2^ in each sub-plot were used. Considering a uniform area in each plot was meant to standardize the data collection method. Sticky traps were spaced 2m apart and placed 10cm above the plant canopy. Hand-picking of insects was done during early morning hours, from 7:30 am to 10:00 am. Insect pests collected were mainly the nymphs and adult stages which were inactive due to the morning cold and dew. At this time, cotton insect pests could not fly easily as observed by Bohmfalk et al. (11).

Insects trapped with sticky traps were removed using kerosene and sieved with fine mesh clothes. The specimens that were sieved and handpicked were preserved in vials containing 70% alcohol. Morphological identification of these specimens to the species level was carried out using published identification keys by Pedigo et al. (12) and Williams (13). Specimen examination was done using a compound microscope in the entomology laboratory at the Tanzania Research Institute (TARI) Ukiruguru, in Mwanza.

### 2.5 Data analysis

The data was organized in Microsoft Excel then subjected to different computations using the Vegan package in R-software. The computations included Shannon diversity, abundance, evenness, and richness indices. To calculate these indices, the Shannon-Weiner diversity index was used for diversity, the total pooled number of insects per week per growth stage was computed for abundance. The Marglef index was used for richness, and the index of Pielou was used for evenness. The formulas for each index were as follows:


(1) Abundance = ∑ni

(2) Shannon (H'=∑i=1SniNlnniN)

(3) $$\text{Species richness index of Margalef }(\text{DMg}=\;(\frac{S-1}{\ln N}))$$

(4) $$\text{Evenness index of Pielou }(\text{J}=\frac{\text{H}}{\ln S}))$$

In these formulae,

*ni* is the number of individuals.

I, is the individual species in the sample;

N, is the total number of individuals in the assemblage;

*S*, is the number of species in the assemblage;

ln, is the natural logarithm.

The computed data was first checked for normality using the Shapiro test. Since the data were not normally distributed, a generalized linear model was used to model abundance and richness using the Poisson distribution error, while diversity and evenness were modelled using binomial distribution error. The models were validated using diagnostic plots (Q-Q plots), and over-dispersion was checked using residual deviance and degree of freedom, following the procedures described by Touchon (14). The Analysis of variance (ANOVA) was derived using the Anova function from the Car package in R. All analyses were performed using R-statistical software. The Tukey’s test was conducted to establish the relationship between the observed and expected values.

## 3 Results

### 3.1 Composition of insets groups

On average, total of 8,585 cotton insect pests were collected for each season, belongs to six families and 12 different species between Nov.2021 and May 2023 (as listed in **Table 1**). Of these 12 species, *Aphis gossypii* was the most abundant, accounting for 17.37% of the total catches. The next three most abundant species were *Amrasca biguttula* (11.42%), *Nezara viridura* (10.7%), and *Bemisia tabaci* (10.68%). These five species collectively accounted for 50.17% of the total catches, while the remaining species accounted for the remaining 49.83% of the catches.

**Table 1:** Number of cotton insect pests collected from established cotton plots.

SN	Family	Species name	Total season 1	Percentage (%)	Total season 2	Percentage (%)	Pooled mean
1	Aphidoidea	*Aphis gossypii*	1,580	17.07	1,402	17.73	1,491
2	Pentatomidae	*Nezara viridura*	1036	11.19	802	10.14	919
3	Pentatomidae	*Caldea degrii*	883	9.54	721	9.12	802
4	Pyrrhoceridae	*Dysdercus cingulatus*	676	7.30	971	12.28	824
5	Cicadellidae	*Amrasca biguttula*	1063	11.48	896	11.33	980
6	Cicadidae	*Cicadellidae Jassidae*	467	5.04	447	5.65	457
7	Aleyorodidae	*Bemisia tabacci*	1195	12.91	639	8.08	917
8	Pseudococcidae	*Phenacoccus solenopsis*	138	1.49	346	4.38	242
9	Miridae	*Lygus lineolaris*	490	5.29	381	4.82	436
10	Thripidae	*Thrips tabaci*	726	7.84	368	4.65	547
11	Noctuidae	*Heliothis armigera Hubner*	392	4.23	636	8.04	514
12	Nolidae	*Earias biplaga*	612	6.61	299	3.78	456
	Total		9,258	100	7,908	100	8,583

### 3.2 Abundance and diversity of cotton insect pests

**Figure 1:**
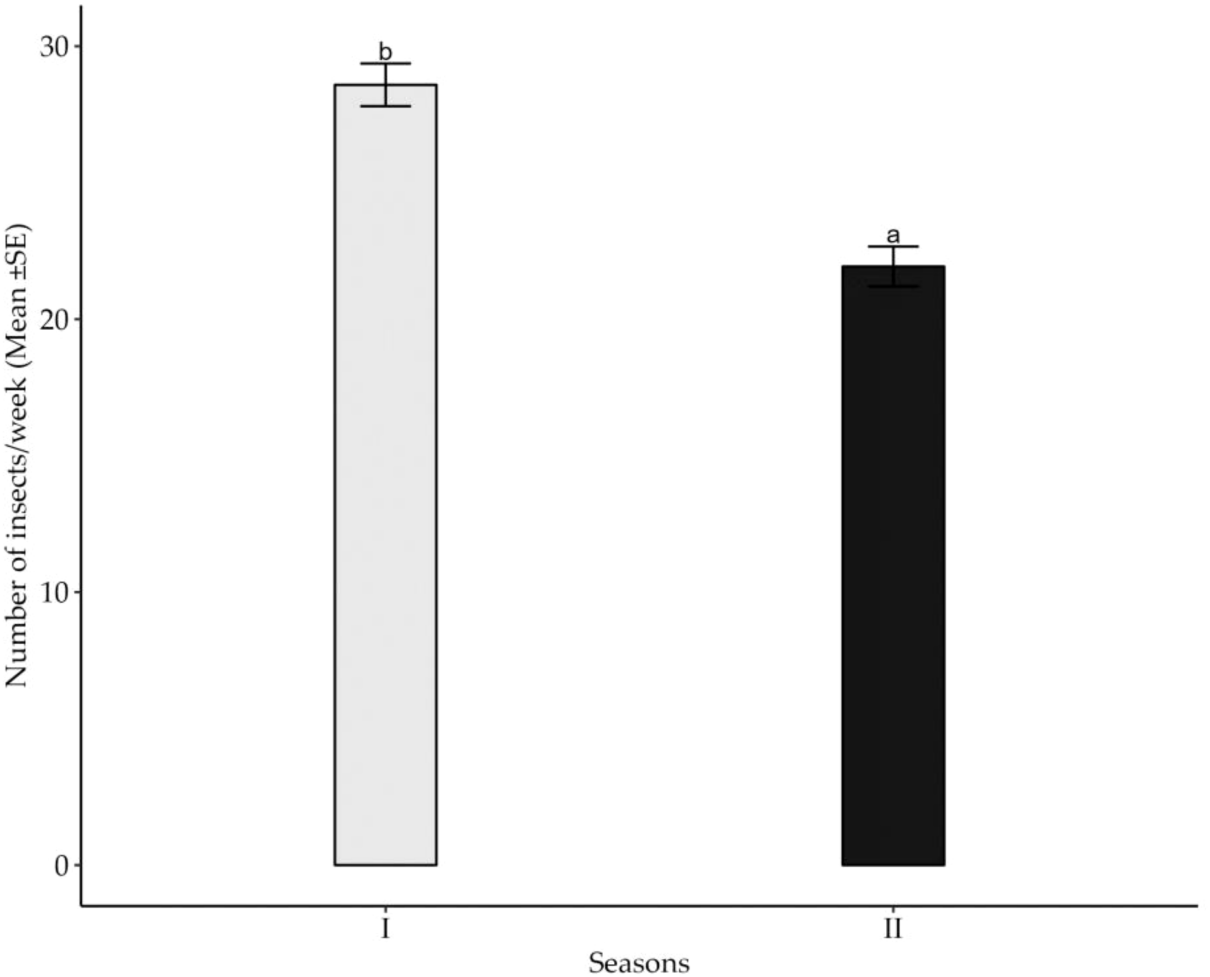
Effects of seasons on mean weekly number of cotton insect pests throughout the seasons across the study areas. The letters above each bar represent the mean separation value with reference to Tukey's Honest Significant Difference (HSD) test with a significance level of α=0.05.

**Figure 2:**
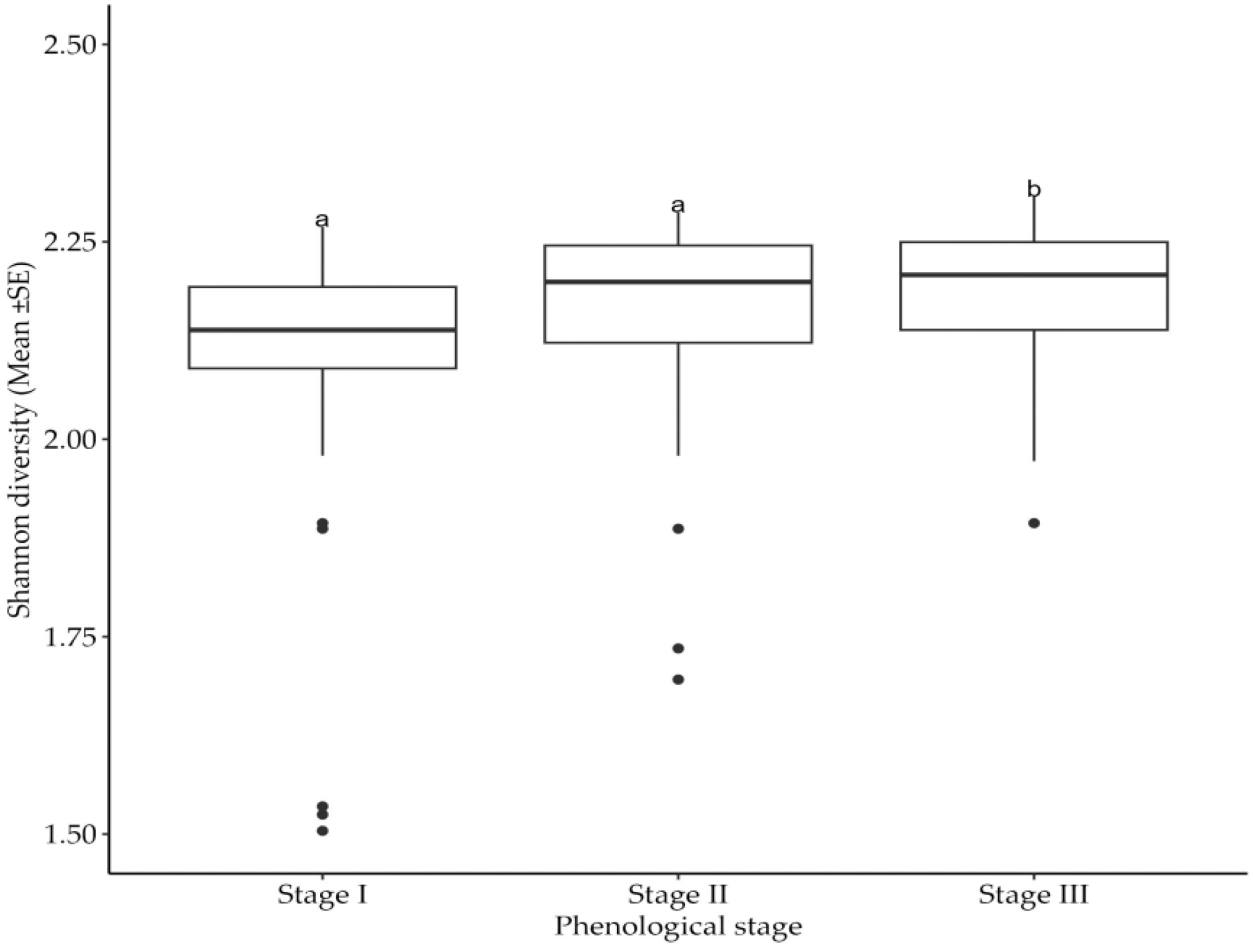
Effects of cotton phenological growth stages on Shannon diversity of cotton insect pest throughout the seasons across the study areas. The letters above each bar represent the mean separation value with reference to Tukey's Honest Significant Difference (HSD) test with a significance level of α=0.05.

The collected data (**Table 2**) indicate the number and diversity of insect pests that infest cotton crops in different phenological growth stages and seasons. The results indicate that the season in which cotton crops are grown significantly (*p*<0.05) impacts on the number of insect pests that infests the crops. Specifically, cotton crops cultivated between November 2021 and May 2022 had a higher number of insect pests than those cultivated between November 2022 and May 2023 (**Figure 1**). Moreover, the cotton phenological growth stages significantly (*p*<0.05) affected the diversity of cotton insect pests. The third stage was found to have a higher diversity of cotton insect pests compared to stages one and two. Nevertheless, phenological stages one and two did not significantly differ in the diversity of cotton insect pests (**Figure 2**).

**Table 2:** ANOVA on the effects of cotton phenological growth stages and seasons on abundance and diversity of insect pests during the 2021/2022 and 2022/2023 seasons.

Variables	Source of variations	Statistics				
		df	SS	MS	F value	P-value
Abundance	Phenological growth stage (Pheno)	2	0.08	0.04	0.25	0.775
	Season (SE)	1	6.54	6.54	43.16	<0.001
	Pheno: SE	2	0.00	0.00	0.01	0.988
	Residual	318	48.18	0.15		
Shannon	Pheno	2	0.68	0.34	16.94	<0.001
	SE	1	0.06	0.06	2.94	0.087
	Pheno: SE	2	0.04	0.02	1.00	0.369
	Residual	318	6.34	0.02		

**Figure 3:**
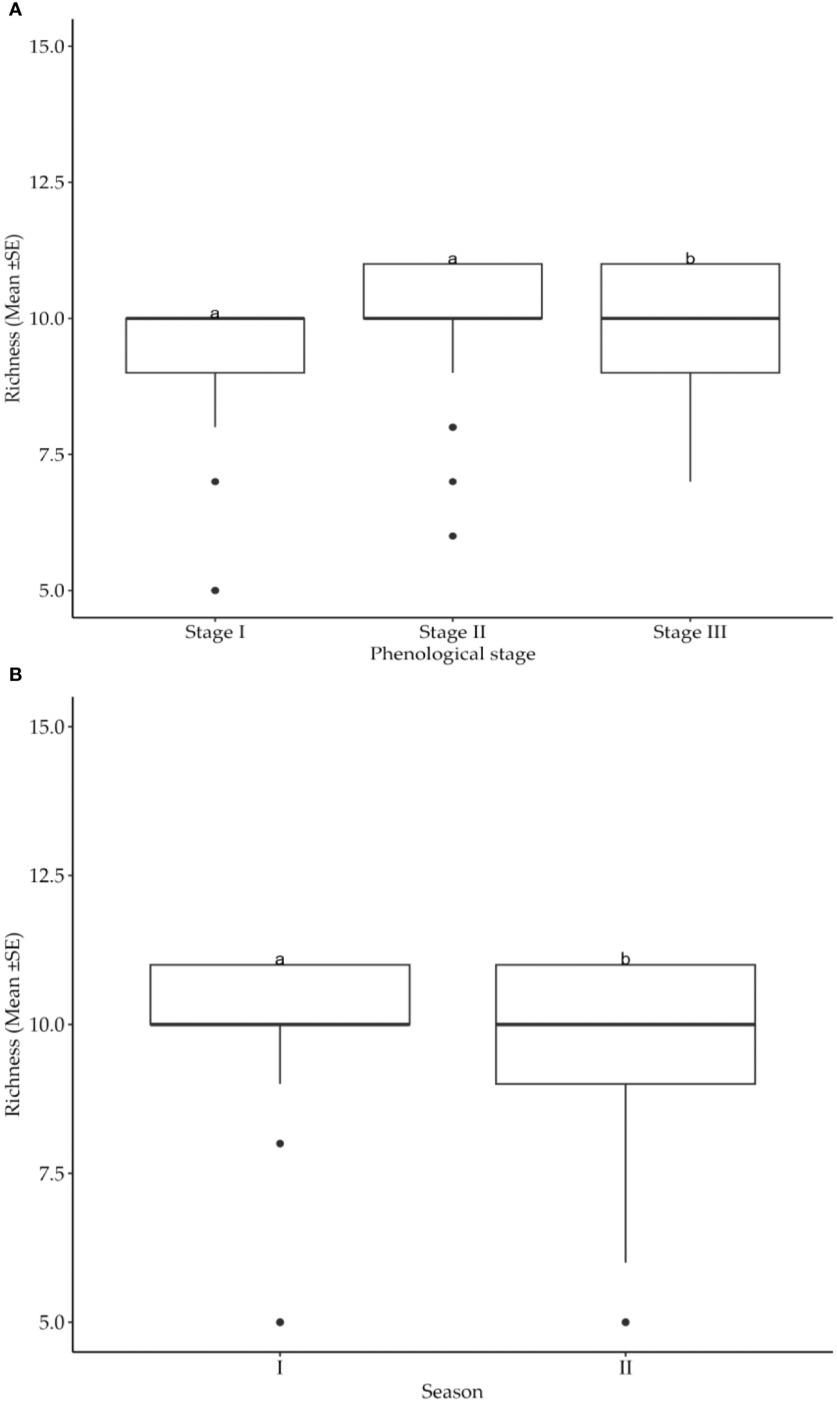
**(A)** Effects of cotton phenological growth stages on cotton insect pest richness throughout the seasons across the study areas. **(B)** Effects of cotton growing seasons on cotton insect pest richness throughout the seasons in the study areas. The letters above each bar represent the mean separation value with reference to Tukey's Honest Significant Difference (HSD) test with a significance level of α=0.05.

Greater diversity in number of insect pest species and their distribution in cotton fields were recorded at different cotton phenological growth stages and seasons (**Table 3**). Suggestively, both growth stages and seasons significantly (*p*<0.05) impacted on the number of insect pest species infesting the cotton crops. Specifically, the growth stage three had higher insect pest richness than stages one and two (**Figure 3A**). Moreover, the insect pest richness was significantly greater during season II compared to season I (**Figure 3B**).

**Table 3:** ANOVA on the effects of cotton phenological growth stages on richness and evenness of insect pests across seasons in the study areas.

Variables	Source of variations	Statistics				
		Df	SS	MS	F value	P-value
Richness	Phonological growth stage (Pheno)	2	52.54	26.27	17.40	<0.001
	Season (SE)	1	10.74	10.74	7.11	0.008
	Pheno: SE	2	2.25	1.12	0.74	0.476
	Residual	318	480.20	1.510		
Evenness	Pheno	2	0.001	0.001	0.572	0.564
	SE	1	0.001	0.001	1.803	0.180
	Pheno: SE	2	<0.001	<0.001	0.022	0.978
	Residual	318	0.249027	<0.001		

### 3.3 Distribution of cotton insect pests

**Figure 4:**
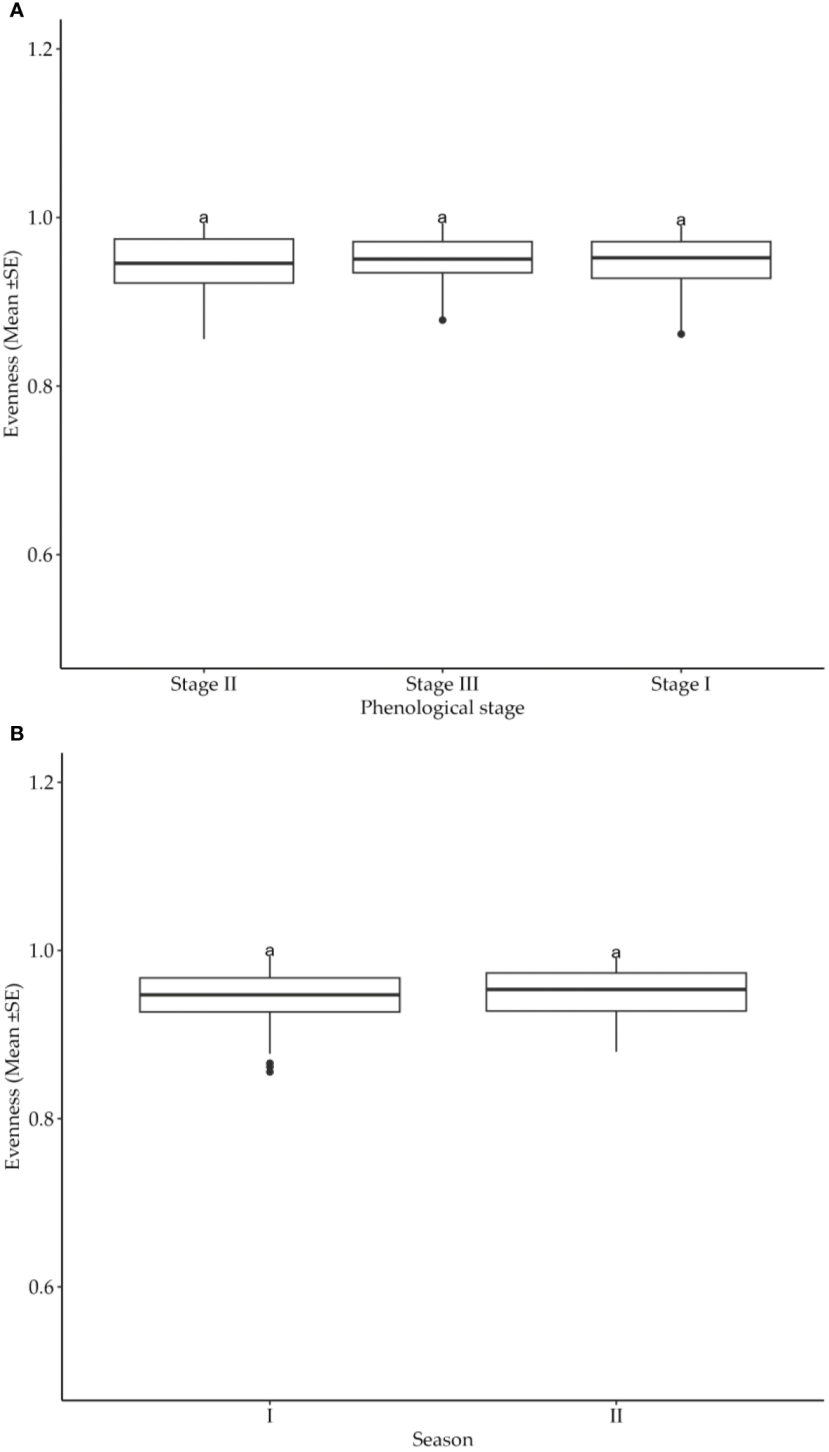
**(A)** Effects of cotton phonological growth stages on cotton insect pest evenness throughout the seasons in the study areas. **(B)** Effects of cotton growing seasons on evenness of cotton insect pests throughout the seasons in the study areas. The letters above each bar represent the mean separation value with reference to Tukey's Honest Significant Difference (HSD) test with a significance level of α=0.05.

The distribution of cotton insect pests was tested in terms of evenness as per phonological growth stages and seasons. Obtained results suggested that neither cotton crop phenological growth stages nor seasons had significant (*p*>0.05) influence on the evenness of insect pest distribution (**Figures 4A, B**). Thus, the pest distribution in the experimental fields was not affected by the crop’s growth stages as well as the production seasons.

### 3.4 Pests population increase with weather parameters’ trend

**Figure 5:**
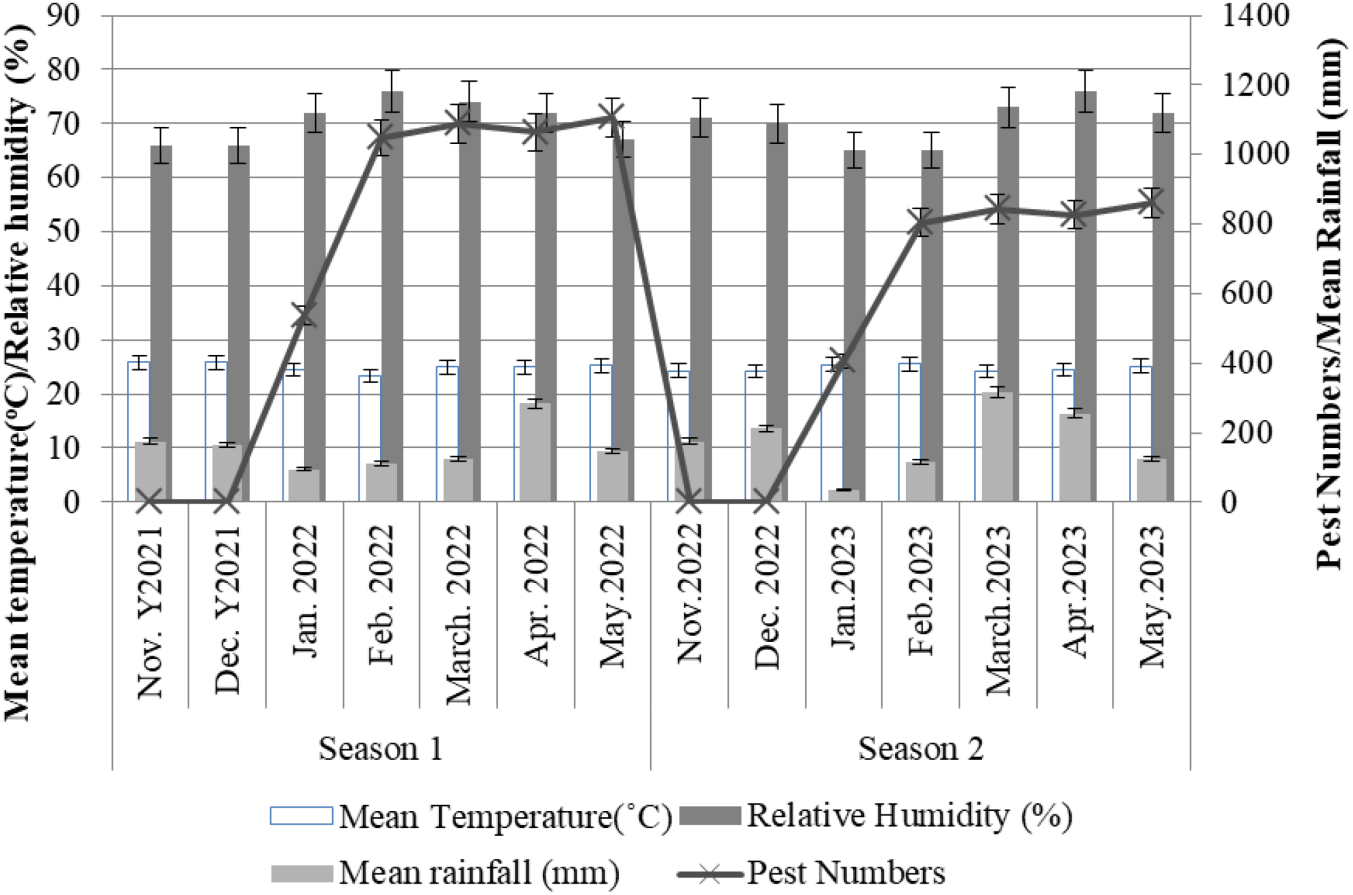
Effects of weather on the population of pest in the study areas.

The number of pests was significantly (*p*<0.05) increased with weather changes. The more the rainfall and relative humidity increase the more pests were increased particularly from January to May every season (**Figure 5**). Off all the months, March and April had high (*p*<0.05) amount of rainfall compared to other months across seasons (**Figure 5**). However, there was no statistical variation between the mean temperatures across the seasons. Season 2 had few pest numbers varying significantly (*p*<0.05) from season 1 (**Figure 5**).

## 4 Discussion

The study delved into the relationship between the insect pest parameters and cotton phenological growth stages and cropping seasons shedding lights on the significance of interactions on insect pest abundance, richness, distribution and diversity. Over 8,500 insects from diverse families and species were collected, highlighting the complex ecological interactions within cotton ecosystems. The dominance of *A. gossypii*, a sap-sucking pest capable of transmitting viral diseases, as the most abundant species is noteworthy (15). The study identified the top four most abundant species to be *A. gossypii*, *A. biguttula*, *N. viridula*, and *B. tabaci.* These pests are a significant problem in agriculture, as they cause extensive yield losses in cotton crops. Other workers (16) reported on the prevalence of these pests in cotton crops, especially in the Western Cotton-Growing Areas of Tanzania.

Characteristics of the season during which cotton crop is grown were to significantly influence the abundance of cotton insect pests. The present study revealed that the cotton crops grown between November 2021 and May 2022 had a higher pest burden than those grown between November 2022 and May 2023. The great abundance of these insect pests in the November 2021 and May 2022 seasons could be attributed to favorable weather conditions particularly the moderate rainfall which favored survival and perpetuation of the insect pest unlike in the 2022/2023 season when excessive rainfall was recorded from February to May. Moderate rainfall, suitable temperatures and relative humidity are known to promote availability of sufficient food resources for insects to thrive while favoring successful reproduction and survival (17). Less rainfall oven associated with higher number of insect pests especially sucking pests (18). It is worth noting that the November 2021 to May 2022 cotton-growing season was characterized by less rainfall than the November 2022 to May 2023 season. The variation noted on pest abundance in season II (2022/2023) where the pest numbers were relatively low was attributed to high rainfall due to the fact that insect pests both sucking and chewing tend to be washed way during heavy rainfall. Similar observations have been reported (18, 19) that the intensity of weather parameters such as rainfall, temperature and relative humidity tends to increase or decrease insect pest abundance. As such, the activities of insect pests in pest endemic areas especially under repeated cultivation of cotton crop in Tanzania are greatly correlated to suitability pf prevailing weather conditions.

On the other hand, the cotton plants itself exhibits different growth stages, each with unique physiological changes. These stages are germination, vegetative growth, flowering and boll formation, which shapes the life cycle of cotton crops (17). Previous studies have shown that insect pest dynamics are not uniform throughout the plant’s life cycle (16, 20). The significance of these growth stages on insect pest diversity has been demonstrated in the present study. It was further established that cotton crops are variably vulnerable to insect pests at different developmental stages. The third growth stage of cotton crops had the highest insect pest abundance and diversity most likely due to increased resource availability and changes in plant physiology (21). These observations align with the report by Johnson et al. (22), who highlighted the complex relationship between plant developmental stages and insect pest diversity. The changes in cotton plant physiology including alterations in secondary metabolites and biochemical processes that occur during the third growth stage which affects the attractiveness of cotton plants to a diverse range of insect species possibly contributed to the observed increase in pest abundance and diversity of insect pests (23).

The impact of cotton growth stages and seasons on Cotton insect pest species richness and distribution in cotton fields was established to be imperative. Insect pest richness was significantly higher at cotton growth stage three compared to stages one and two, indicating a possible link between cotton plant developmental stages and insect diversity. Similar observations have been reported by other workers (24, 25) whereby specific growth stages have been found to be important in shaping insect communities in the agricultural environments. Several factors could explain the observed increase in insect species richness during growth stage three. The availability of resources, such as developing bolls and a profusion of floral resources, is likely to attract a wide range of insect species (26, 27). Changes in plant physiology, such as modifications in secondary metabolites and volatile organic compounds, may also make cotton plants more appealing to a broader range of insect pests during this critical developmental stage (27, 28).

The pest population increase may have been contributed by the abundance of habitats through the rapid emerging thicket forests at rain season in particular. In wet season several vegetation emerges which appear to be the common habitats for insect pests and water is easily accessible by the insects implying that the breeding environments for them is increasing as similarly reported by Brown and Smith (28). However, relative humidity might have led to atmospheric cooling as the mean temperature was less than 30°C across seasons.

## 5 Conclusion

This study provides valuable insights into the abundance, diversity, distribution and species richness of cotton insect pests in areas under repeated production of the crop. Cropping seasons and crop phenological developmental stages were found to significantly affect the abundance, diversity and distribution of cotton pests. These findings are crucial for developing sustainable pest management strategies at different crop growth stages particularly during an era of climate change that has brought about changes in seasons characteristics. The continuous threat these pests pose requires repeated research to uncover local nuances in their distribution dynamics. This will enable more precise and effective pest control strategies in cotton producing areas of Tanzanian and elsewhere with similar production circumstances.

## Data Availability

The original contributions presented in the study are included in the article/supplementary material. Further inquiries can be directed to the corresponding author.

## References

[1] Khalil H Raza ABM Afzal M Aqueel MA Khalil MS Mansoor MM. Effects of plant morphology on the incidence of sucking insect pests complex in few genotypes of cotton. J Saudi Soc Agric Sci. (2017) 16:344–9. doi: 10.1016/j.jssas.2015.11.003

[2] Mrosso F Mwatawala M Rwegasira G. Effect of lambdacyahalothrin 5% EC on C*cheilomenes lunata, Cheilomenes sulphurea* and *Cheilomenes propinqua* (colleoptera: coccinellidae) predators of cotton Aphids (*Aphis gossypii*) (Homoptera: Aphididae) in Eastern Tanzania. J Entomol. (2014) 11:306–18. doi: 10.3923/je.2014.306.318

[3] Naeem-Ullah U Ramzan M Bokhari SHM Saleem A Qayyum MA Iqbal N. Insect pests of cotton crop and management under climate change scenarios. Enviro climate Plant vegetation Growth. (2020), 367–96. doi: 10.1007/978-3-030-49732-3_15

[4] Unsar NU ur Rahman MH Fahad S Shafqat S. Insect pests of cotton crop and management under climate change scenarios. In: Environment, Climate, Plant and Vegetation Growth. Switzerland: Springer Publishers Ltd, Springer Nature Switzerland (2020). p. 367–96.

[5] Oerke EC Dehne HW Schönbeck F Weber A. Crop Production and Crop Protection: Estimated Losses in Major Food And Cash Crops. Germany: Elsevier (2012). p. 829.

[6] Malik AK Mansoor S Saeed NA Asad S Zafar Y Stanely J. Development of CLCV Resistance Cotton Varieties through Genetic Engineering. Punjab, Pakistan: Director of Agricultural Information (1995). 3pp.

[7] Nawaz B Naeem M Malik TA Muhae-Ud-Din G Ahmad Q Sattar S. A review about cotton leaf curl viral disease and its control strategies in Pakistan. Int J of. Innov Applied Agricult Res. (2019) 3:132–47. doi: 10.29329/ijiaar.2019.188.13

[8] Ballari HS Udikeri SS. Persisting resistance of *helicoverpa armigera* (Lepidoptera: noctuidae) to pyrethroid, organophosphate and carbamate insecticides. Artic Pakistan J Zool. (2021) 8:171–82. doi: 10.17582/journal.pjz/20200822140857

[9] Johnson L. Assessing the Relationship Between Weather Parameters on Cotton (2013). Available online at: http://dspace.cbe.ac.tz:8080/xmlui/handle/123456789/769 (accessed January 12, 2024).

[10] Saidia PS Mrema JP. Effects of farmyard manure and activated effective microorganisms on rain-fed upland rice in Mwanza, Tanzania. Organ Agric. (2016) 7:83–93. doi: 10.1007/s13165-016-0154-6

[11] Bohmfalk GT Frisbie RE Sterling WL Metzer. RB Knutson AE. Identification, Biology and Sampling of Cotton insects (2011). Available online at: http://www.soilcropandmore.info/crops/CottonInformation/insect/B-933/b-933.htm (accessed December 14, 2023).

[12] Pedigo LP Rice ME Krell RK. Entomology and pest management. 6th Edition Vol. 60047-9580. Illinois, USA: Waveland Press (2021) p. 638–42.

[13] Williams DJ. Mealybugs of southern Asia Vol. 5. Malaysia: Natural History Museum/Southdene Sdn Bhd (2004) p. 1–896 18–2.

[14] Touchon JC. Applied statistics with R: a practical guide for the life sciences Vol. 59. UK: Oxford University Press (2021) p. 137–49.

[15] Cheng S Li R Chen Z Ni J Lv N Liang P. Comparative susceptibility of Aphis gossypii Glover (Hemiptera: Aphididae) on cotton crops to imidacloprid and a novel insecticide cyproflanilide in China. Ind Crops Prod. (2023) 192:116053. doi: 10.1016/j.indcrop.2022.116053

[16] Lusana MS Tarimo AJP Mulungu LS. Abundance and composition of cotton insect species in the western cotton growing area (WCGA) in Tanzania. J Entomol Zool Stud. (2019) 7(6):1087–92.

[17] Li N Li Y Biswas A Wang J Dong H Chen J. Impact of climate change and crop management on cotton phenology based on statistical analysis in the main-cotton-planting areas of China. J Clean Prod. (2021) 298:126750. doi: 10.1016/j.jclepro.2021.126750

[18] Sharma HC. Climate change effects on insects: implications for crop protection and food security. J Crop improve. (2014) 28:229–59. doi: 10.1080/15427528.2014.881205

[19] Skendžić S Zovko M Živković IP Lešić V Lemić D. The impact of climate change on agricultural insect pests. Insects. (2021) 12:440. doi: 10.3390/insects12050440 34066138 PMC8150874

[20] Allen KC Luttrell RG Sappington TW Hesler LS Papiernik SK. Frequency and abundance of selected early-season insect pests of cotton. J Integr Pest Manag. (2018) 9(1):1–11. doi: 10.1093/jipm/pmy010

[21] Robertson A McDonald RA Delahay RJ Kelly SD Bearhop S. Resource availability affects individual niche variation and its consequences in group-living European badgers Meles meles. Oecologia. (2015) 178:31–43. doi: 10.1007/s00442-014-3202-5 25656581

[22] Huang J Hao H. Detecting mismatches in the phenology of cotton bollworm larvae and cotton flowering in response to climate change. Int J Biometeorol. (2018) 62:1507–20. doi: 10.1007/s00484-018-1552-0 29752540

[23] Singh V Mandhania S Pal A Kaur T Banakar P Sankaranarayanan K. Morpho-physiological and biochemical responses of cotton (Gossypium hirsutum L.) genotypes upon sucking insect-pest infestations. Physiol Mol Biol Plants. (2022) 28:2023–39. doi: 10.1007/s12298-022-01253-w PMC9789232 36573153

[24] Mohapatra MM Singh DC Gupta PK Chandra U Patro B Mohapatra SD. Seasonal incidence of major insect-pests on blackgram, Vigna mungo (Linn.) and its correlation with weather parameters. Int J Curr Microbiol Appl Sci. (2018) 7:3886–90. doi: 10.20546/ijcmas

[25] Ashra H Nair S. Trait plasticity during plant-insect interactions: From molecular mechanisms to impact on community dynamics. Plant Sci. (2022) 317:111–88. doi: 10.1016/j.plantsci.2022.111188 35193737

[26] Nemade PW Budhvat KP Wadaskar PS. Population dynamics of sucking pests with relation to weather parameters in bt cotton in Buldana 77 district, Maharashtra, India. Int J Curr Microbiol Appl Sci. (2018) 7:1–7. doi: 10.20546/ijcmas.2018.701.075

[27] Jasrotia P Kumari P Malik K Kashyap PL Kumar S Bhardwaj AK. Conservation agriculture based crop management practices impact diversity and population dynamics of the insect-pests and their natural enemies in agroecosystems. Front Sustain Food Syst. (2023) 7:1173048. doi: 10.3389/fsufs.2023.1173048

[28] Brown GH Smith PQ. The interplay of resource availability and altered plant physiology in shaping insect pest communities. J Appl Ecol. (2017) 35(1):89–104.

